# DBSCAN Spatial Clustering Analysis of Urban “Production–Living–Ecological” Space Based on POI Data: A Case Study of Central Urban Wuhan, China

**DOI:** 10.3390/ijerph19095153

**Published:** 2022-04-23

**Authors:** Xiaoqiang Tu, Chun Fu, An Huang, Hailian Chen, Xing Ding

**Affiliations:** 1 School of Management, Nanchang University, Nanchang 330031, China; dingxinsusan@163.com; 2 School of Architecture, Tsinghua University, Beijing 100084, China; hhanner@163.com; 3 School of Science and Technology, Gannan Normal University, Ganzhou 341000, China; chenhailian2008@163.com

**Keywords:** POI, production–living–ecological, spatial planning, DBSCAN, Wuhan

## Abstract

As urban spatial patterns are the prerequisite and foundation of urban planning, spatial pattern research will enable its improvement. The formation mechanism and definition of an urban “production–living–ecological” space is used here to construct a classification system for POI (points of interests) data, crawl POI data in Python, and DBSCAN (density-based spatial clustering of application with noise) to perform cluster analysis. This mechanism helps to determine the cluster density and to study the overall and component spatial patterns of the “production–living–ecological” space in the central urban area of Wuhan. The research results are as follows. (1) The spatial patterns of “production–living–ecological” space have significant spatial hierarchical characteristics. Among them, the spatial polarizations of “living” and “production” are significant, while the “ecological” spatial distribution is more balanced. (2) The “living” space and “production” space noise points account for a small proportion of the total and are locally clustered to easily become areas with development potential. The “ecological” space noise points account for a large proportion of the total. (3) The traffic accessibility has an important influence on the spatial patterns of “production–living–ecological” space. (4) The important spatial nodes of each element are consistent with the overall plan of Wuhan, but the distribution of the nodes for some elements is inconsistent. The research results show that the POI big data can accurately reveal the characteristics of urban spatial patterns, which is scientific and practical and provides a useful reference for the sustainable development of territorial and spatial planning.

## 1. Introduction

In recent years, the rapid development of industrialization and urbanization has led human activities to cause significant disturbances in land use, which has squeezed the agricultural and ecological space to produce intensified contradictions between agriculture, ecology, and urbanization with the sustainable development and utilization of urban land space that has been severely impacted [1]. Urban space is the operational carrier of urban societies, economies, politics, culture, and other elements [2]. Functional areas formed by various urban activities constitute the basic framework of urban spatial structures. However, excessive social and economic activities have resulted in the inefficient use of production space, poor livability, and severe compression of the ecological space (i.e., “production–living–ecological” space), different spaces having incoordination problems in terms of scale ratios, spatial configuration, and functional integration [3]. Spatial patterns are not only the basis for dividing functional areas for urban planning but also the basis for testing whether the positioning of functional areas in urban planning is reasonable [4,5,6,7,8]. Therefore, research on spatial patterns of the urban “production–living–ecological” space not only contributes to the strategic improvement of space optimization but also improves of urban planning and promotes the development of urban spaces toward being “intensive and efficient, suitable for livability, and good environment”; enhancing the ability of cities to cope with emergencies (such as COVID 19, etc.); and remaining sustainable, all of which are of great importance.

Research on the spatial distribution patterns of “production–living–ecological” space has attracted significant attention, and scholars have performed detailed research over different spatial scales. From a macro perspective, Zhang et al. [9] constructed a classification system for “production–living–ecological” space based on the main functions of land and extracted the associated spatial distribution patterns for China. Liu et al. [10] established a spatial classification system for “production–living–ecological” space based on the classification of land use status, which revealed the spatial patterns in China from 1990 to 2010. They found that the production space is distributed primarily on the southeast side of the Hu Huanyong Line, the living space is concentrated in major cities and urban agglomerations, and the ecological space is distributed on the northwest side of the Hu Huanyong Line. Based on survey data, Bai et al. [11] used a multi-dimensional econometric model to explore the spatial differentiation characteristics of the “production–living–ecological” space of the Jianghuai urban agglomeration. Jin et al. [12] constructed the “production–living–ecological” spatial–functional index using land-use data and discussed the spatial differentiation patterns of the Fujian Triangle City Group. Liu et al. [3] analyzed the spatial patterns of “production–living–ecological” space in Chinese cities using remote sensing data from land use and statistical yearbook data.

At the meso-scale, Cui et al. [13] used national land survey data and grid analyses to reveal the spatial distribution pattern of the “production–living–ecological” space in Hubei Province. Zhang et al. [14] used remote sensing monitoring data from land-use status and raster methods to evaluate the heterogeneity of Hainan Island’s “production–living–ecological” space. At the micro-scale, Wang et al. [15] used land-use change survey data and statistical yearbook data to construct a spatial-function coupling and coordination model of Chongqing’s rural “production–living–ecological” space, which revealed the spatiotemporal differentiation characteristics of coupling and coordination. Jiao et al. [16] analyzed the spatial distribution patterns of the “production–living–ecological” space in Wuyuan County using the landscape pattern index with remote sensing data from land use. Thus, for some time, traditional land survey data, remote sensing monitoring data, and socio-economic data have supported research on the spatial patterns of “production–living–ecological” space, but these methods have strong spatial scale dependencies. In addition, traditional data come from government departments, which are relatively difficult to obtain and have a long update cycle. This increases the difficulty of meeting the accuracy requirements to dynamically describe the characteristics and laws of urban spatial layouts [17].

Geospatial big data as represented by POI points provide new concepts for urban spatial structure research [18] The POI data use structures such as points, lines, and areas to abstractly express geographic entities, such as supermarkets and schools [19]. Spatial statistical analysis methods are widely used in the analysis of spatial patterns in urban facilities [20], the evaluation of urban human settlements [21], and the division of urban functional spaces [22]. For urban facilities, the application results of commercial retail space distributions [23], service industry spatial layouts [24], and public facility spatial patterns are very rich. For urban human settlement environmental evaluations, the information carried by POI data is used to evaluate the human–land relationship between the accessibility and convenience of urban facilities, as well as to evaluate the built environment of communities. For urban functional space division, the entity city POI data are organized based on the classification system; the spatial density of the classified POI data is calculated; and the urban spatial characteristics and degree of aggregation are analyzed to identify urban spaces, such as urban functional and central areas. As research continues to deepen, the complement of POI big data and other multi-source data will help explore the human-land coupling relationships and their laws in-depth. Although POI data have been widely used to research humanities and economic geography, there are few studies on its application to the spatial distribution patterns of urban “production–living–ecological” space.

This paper is based on the POI data of Wuhan’s central city area and uses density-based spatial clustering for applications with the noise (DBSCAN) clustering algorithm using Python and ArcGIS software to analyze the spatial patterns of the “production–living–ecological” space. The research results are compared with the latest urban planning in the central urban area of Wuhan, to determine whether there is a misalignment of functional positioning and to put forward optimization suggestions accordingly, providing a useful reference for the optimization of urban “production–living–ecological” space and the improvement of urban planning. This paper overcomes the drawbacks of traditional official data, fills the gap in the application of POI data to the study of urban “production–living–ecological” space, and further broadens the horizons of urban “production–living–ecological” spatial pattern research.

The first part of the article is the introduction, the second part is the introduction of the formation mechanism and concept of the “production–living–ecological” space, the third part is the introduction of the methods used in the article, the fourth part is the analysis of the research results, the fifth part is the discussion, and the sixth part is the in conclusion.

## 2. The Formation Mechanism and Concept of “Production–Living–Ecological” Space

With the rapid advancement of industrialization and urbanization, the territorial spatial pattern has been suffering an unprecedented shock: agricultural space and ecological space are extruded; the contradiction between humans, the environment, and resources is accelerating; the regional development gap is widening gradually; there is extensive and serious land use [1,25]; and the sustainable development of territorial spaces is facing severe challenges. These factors all need to be optimized again. Therefore, the Chinese government has successively introduced policies on territorial spatial planning. In 2000, the National Development and Reform Commission of China required local governments to ensure the coordination of space, people, resources, and the environment when planning industrial layouts. In 2008, the Outline of the National General Plan for Land Use (2006–2020) clearly required that the proportion of ecological land should be increased, and the land used for “production–living–ecological” space should be rationally coordinated. In 2010, the National Plan for Major Functional Zones required that the carrying capacity of regional resources and environment be assessed; production space, living space, and ecological space be planned in an overall way; and the proportion of ecological space be enlarged. In the report of the 18th CPC National Congress in 2012, Chinese President Xi Jinping clearly put forward the overall requirements for ecological civilization construction of “intensive and efficient production space, livable and moderate living space, and picturesque living space” [26]. Since then, “production–living–ecological” space has attracted high attention from Chinese government departments and academic circles, making it the overall goal of territorial spatial planning.

**Figure 1:**
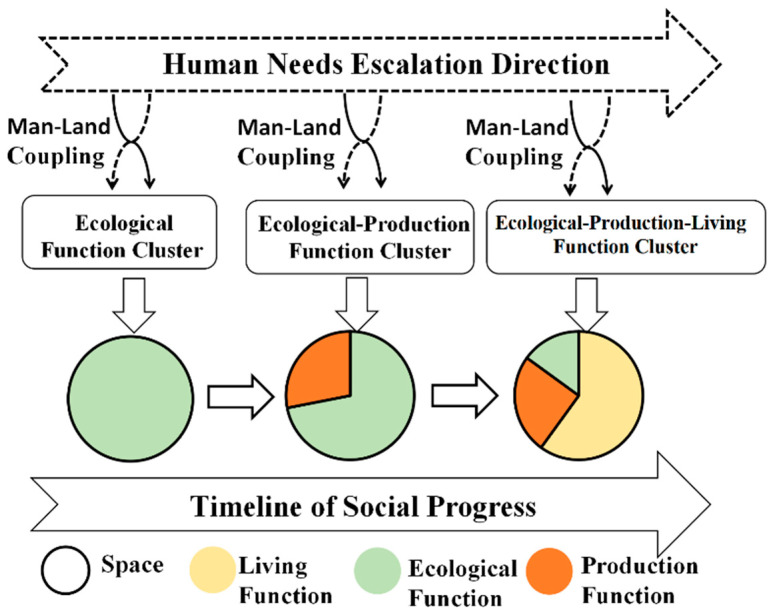
Mechanism of formation for the “production–living–ecological” space [33].

The “production–living–ecological” space is based on multi-functional land use, which originates from the coupling of humans and land [27], as seen in Figure 1. The land contains multiple functions [28], and the SENSOR project claims that land can provide humans with three major functions: society, economy, and environment [29]. De Groot et al. [30] divided land into the three major functions of society, economy, and ecological environment, and Li et al. [31] divided land space into the three major functional spaces of ecological, production, and living. Land is the material basis and space place for human survival [32]. In the early stages of human society, food came from nature and lived in nature. Humans had an insufficient ability to transform nature dynamically, and land functions were more manifest as ecological functions. With the emergence of the agricultural society, gifts of nature could meet the growth of human survival and living needs. Humans actively transformed the land, and agricultural functions were developed, which were gathered to form the agricultural function space (that is, the production function space). With the improvement in external survival and living environments, human needs continually increased, and the demand for diversity made the coupling between humans and land more diversified. As humankind entered industrial society, the multi-functionality of land use was fully developed, and the gathering of living functions formed the living function space. Thus, under the influence of the regional leading functions, a “production–living–ecological” space was formed.

Different scholars interpret the concept of “production–living–ecological” space from different perspectives. From the functional perspective, Li Guangdong et al. [31] believed that “production–living–ecological” space is three functional spaces of ecology, production and living, and is the product of the synergistic coupling of the natural system and the social economic system. Huang Jinchuan et al. [34] also interpreted it from a functional perspective but emphasized more that space is the area providing major functions. Zhang Hongqi et al. [9] and Zhu Yuanyuan et al. [35] interpreted “production–living–ecological” space from the perspective of land use properties, believing that production space provides production and operation services for human beings; ecological space provides environmental fundamental space for human survival and development; and living space provides humans with space for living, entertainment, education, and so on. From the perspective of ecological civilization, Liu Yan [36] believed that living space is a space with strong living functions such as food, clothing, housing, travel, entertainment, and education; production space is an activity space for producing commodities; and ecological space is a space for activities to sustain life. Huang An et al. [27] integrated the formation mechanism of “production–living–ecological” space and believed that production space is space featured production function, mainly to provide human with material and non-material products and production-related services; living space features living functions, meeting humans’ different demands in habitation, consumption, entertainment, health care, education and so on; and ecological space is a space dominated by ecological functions, providing ecological products and services, undertaking the formation of ecological systems and ecological processes, and maintaining the natural conditions and their utilities for human existence.

Via a comprehensive review of definitions of different scholars, combined with a formation principle of “production–living–ecological” space in cities, this paper holds that urban production space is associated with industrial structure, serving as the functional space mainly for industrial products; agricultural products; and the production of service products, including commerce and business spaces (such as companies and spaces for financial services), industrial spaces (such as factories and industrial parks, warehousing and logistics, and space for auto-related services), transportation space (such as urban roads, subways, parking lots, airports, bus stops, ports and docks, etc.). It also holds that urban living space is the functional space related to carrying and guaranteeing the living and housing of human being, by providing human beings with functional spaces mainly for living, entertainment, health, culture, education, shopping, and public services. These include living spaces and commercial service spaces (such as retail monopoly, supermarket, accommodation, catering, etc.), public space (such as hospitals, schools, museums, public facilities, etc.) and management space (such as government, social institutions, etc.). Furthermore, this paper holds that urban ecological space is a functional space related to the natural and artificial environments, providing ecological products and services for human beings, including green land spaces (such as parks, squares, zoos, botanical gardens, etc.) and space for places of interest (such as scenic spots, abbeys, Taoist temples, world heritage sites, etc.).

## 3. Materials and Methods

### 3.1. Study Area and Data Source

#### 3.1.1. Study Area

**Figure 2:**
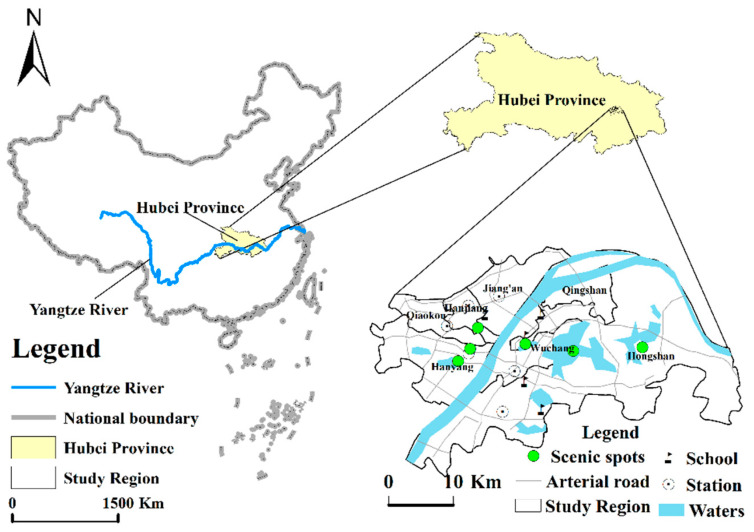
Map showing the location of Wuhan city center. Location map of the study area.

This paper selects the central urban areas of Wuhan, China (Jiang’an District, Jianghan District, Qiaokou District, Hanyang District, Wuchang District, Hongshan District, and Qingshan District) as the research scope (see Figure 2). Wuhan is the capital city of Hubei Province in China, with geographic coordinates of 29°58′–31°22′ N and 113°41′–115°05′ E. Wuhan is in the east of the Jianghan Plain and the central reaches of the Yangtze River, which meets its largest tributary, the Han River, in the city. The traffic conditions are also considered to be excellent. Therefore, Wuhan is the central city of China’s comprehensive transportation system. As a central city of China’s economic geography, Wuhan is accelerating the completion of five key functional areas: the national economic center, the regional financial center, the national science and technology innovation center, the trade and logistics center, and the international exchange center. For the national economic center, the central city promotes the construction of the Hankou Riverside Business District, Wuchang Riverside Business District, and Yangchun Lake Business District and plans for key functional areas, such as the Qingshan, Hanyang, Chenjiaji, and Baisha Binjiang Districts, to lead the upgrades for modern service industries, development zones, and new urban areas. This supports the development of advanced manufacturing industries focused on the National Memory Base, the National Aerospace Industry Base, the National New Energy and Intelligent Connected Vehicle Base, the National Cyber Security Talent and Innovation Base, and the Big Health Industry Base.

For the national science and technology innovation center, the “Da Wuchang + Yangtze River New District” as the core cluster area continues to promote the construction of key functional areas, such as East Lake Science City, Optics Valley Science Island, and Hongshan University City. This cultivates and develops the Yangtze River New District science and education city and carries comprehensive functions of the science center and the science and technology innovation center. For the national trade and logistics center, with the Greater Hankou area as the core agglomeration area, the focus on promoting the construction of key functional areas (such as the Hanzheng Street Central Service Area and Hankou North Commercial Area), carrying the functions of commerce and consumption centers, and enhancing key functions (such as Wuhan Aviation City and Yangluo International Port District) creates the conditions of an integrated transportation hub. For the international exchange center, the Greater Hanyang area is the core gathering area and focuses on promoting the construction of key functional areas, such as Guibei and the Sino-French eco-city, and hosting functions, such as international competitions and cooperation. For the regional financial center, the focus is on the development of the headquarters of the financial cluster area, using key functional areas, such as the Wuhan Financial City, Optics Valley Central City, Wuchang financial street, and Hanyang fund base, as the carriers [37].

The base map data comes from the geographic information resource catalog service system of China (1:1 million, https://www.webmap.cn/commres.do?method=result100W, accessed on 13 November 2021).

#### 3.1.2. Data Source

The data involved in this research include the following. (1) POI data were points of interest within the city center of Wuhan as sourced from the Auto Navigation Map in 2019. Python was used to write programs for crawling, which was followed by ArcGIS spatial analysis methods. A total of 321,716 POI data points was obtained after extracting the POI data from the research area and performing data preprocessing (removing data redundancy), as seen in Table 1. These data included eight attributes (name, type, address, longitude, latitude, administrative area, address, and administrative area code) and 19 major categories, with each containing multiple sub-categories. (2) The boundary map of the study area comes from the National Basic Geographic Information System database.

### 3.2. Methods

The research concept of this article is given as follows. First, a POI classification system was built based on the formation mechanism and definition of the urban “production–living–ecological” space. Second, the collected POI data were preprocessed to eliminate redundant and out-of-range data. Third, Python (Corporation for National Research Initiatives, Reston, VA, USA) was used to write programs, and DBSCAN clustering iterative calculations were performed on the three types of POI data of production, living, and ecology. Appropriate minimum points (MinPts) and Eps parameters (neighborhood parameters ε) were selected, and ArcGIS (Environmental Systems Research Institute, Redlands, CA, USA) was used to calculate the density of each cluster to analyze the overall spatial patterns of “production–living–ecological” space. Fourth, DBSCAN clustering iterative operations were performed on the element layer, appropriate MinPts and ε were selected, ArcGIS was used to calculate the density of each cluster, and the spatial patterns of each element layer were analyzed. Fifth, a scientific reference was provided to optimize the “production–living–ecological” spatial structure in the downtown area of Wuhan.

#### 3.2.1. POI Data Classification

The above-mentioned understanding of the formation mechanism and definition of the urban “production–living–ecological” space was used to draw lessons from Hu et al.’s [38] urban spatial classification system. The principles of consistency and universality are as follows for the POI data to construct a classification index system, as seen in Table 1. The indicator system consists of three layers: target, criterion, and element. The target layer includes the three major functional spaces of production, living, and ecology. The criterion layer is a sub-category of the three major functional spaces with a total of eight criterion layers, which are composed of several element layers and give a total of nineteen layers. Each element layer corresponds to a detailed category of the element.

#### 3.2.2. DBSCAN Clustering Algorithm

The DBSCAN algorithm is a density-based spatial data clustering method [39]. The advantages of the DBSCAN algorithm are that it can effectively solve problems from large amounts of data, the overlapping and covering from points of interest, and its fast clustering speed, which can effectively handle noise points and divide high-density areas into clusters to form easier-to-recognize spatial clusters [40]. The combination of DBSCAN cluster analysis and visualization software such as ArgGIS can process data in a more accurate and intuitive way, overcoming the shortcomings of traditional network kernel density algorithms. Compared with the K-means clustering method, which is only suitable for convex sample sets, the DBSCAN clustering method can be applied to both convex and non-convex sample sets. In addition, compared with distance-based and hierarchical division-based clustering methods, the DBSCAN clustering method can consider the similarity of spatial and attribute data at the same time, find spatial clusters of arbitrary shapes, and better identify outliers, and it does not require input data to make any assumptions about the distribution. Therefore, this paper selects the density-based DBSCAN clustering method to identify the spatial distribution pattern of urban “production–living–ecological” space.

The DBSCAN algorithm describes the tightness of sample distributions based on a set of neighborhood parameters ε and MinPts, where ε is the radius of the neighborhood, and MinPts is the threshold for the number of smallest objects within the radius of the neighborhood. Given a data set D = {x1, x2, ..., xm}, the following concepts are defined [41]:

ε-neighborhood: For xj ∈ D, xj is taken as the center of the circle with ε as the radius of the neighborhood (ε > 0), and the data samples are set as those that fall into the space of the neighborhood.

Core object: If at least MinPts sample data are included in the ε-neighborhood of xj, xj is the core object of the neighborhood.

Direct density-reached: If xj is in the ε-neighborhood with xi as the core object, xj is considered to be directly reached by the xi density.

Density-reachable: If a group of objects x1, x2, x3, ..., xn for any i ∈ [1, n − 1] and x (i + 1) directly reaches the density of xi, the density of xn and x1 are considered reachable.

Density-connected: If there is an object xk so that the densities of both objects xi and xj are reachable with the object xk, then xi and xj are considered connected in density.

Noise (outlier) point: If there is an object xj, and there is no density-connected point in its ε-neighborhood, then xj is the noise (outlier) point.

**Figure 3:**
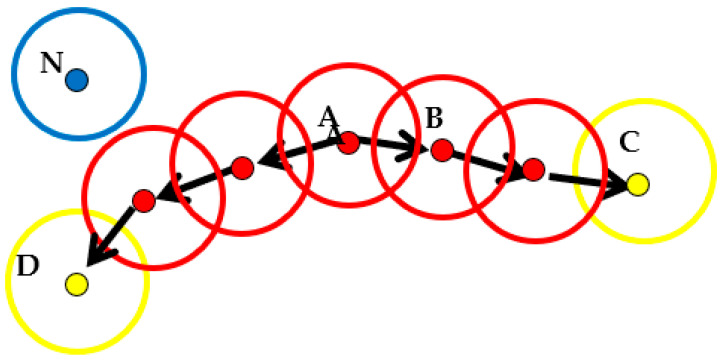
Schematic diagram of the DBSCAN clustering algorithm implementation. Where N is the noise (outlier) point, the circular solid line is the ε-neighborhood, A is the core object, B is directly reached by the density of A, C and D are reachable by the density of A, and the densities of C and D are connected.

The implementation principle of the DBSCAN algorithm is shown in Figure 3. First, all the core objects in the data sample are determined based on the given core parameters (ε, MinPts). The core objects are determined as being a core object set or not, a core object is chosen as a seed, and the center of a circle of radius ε is made to find all samples that are not less than MinPts and connected in density. These samples form a cluster with a high density, and a core object is selected from the re-updated core object set as a seed to generate the next high-density cluster. The above process is repeated until the core object set is empty, which forms the final cluster. If an object is not densely accessible to other objects, it is considered a noise (outlier) point.

From the above analysis, the core parameters of the DBSCAN algorithm are the ε and MinPts, where the quality of the core parameter settings determines the quality of the clustering results [42]. The ε is determined based on the Euclidean distance between objects and the distance in the descending order of K. A set that is too large easily leads to relatively large clustering results. A set that is too small readily regards sparse clusters as noise points. As the POI data of the living and production spaces are dense and concentrated in the core area of the city center, large clusters are formed regardless of the parameters selected. Therefore, the selection criteria for this article are as follows: (1) the cluster evaluation coefficient is [–1, 1] and is a positive value, and (2) the boundary between each cluster is obvious.

#### 3.2.3. Cluster Density Calculation

This article uses the ArcGIS software to calculate the POI data distribution density for each cluster. Step 1 is to generate the triangulated irregular net (TIN) and to use Delaunay triangulation to create the irregular triangulation. Step 2 is to depict the TIN using the PERIMETER_ONLY method to make the irregular triangulation more closely fit the distribution boundary of each cluster. Step 3 is to generate a polygon area feature by outputting the TIN generated in the previous step as a polygon area feature that is closer to the distribution boundary. Step 4 is to calculate the clustering distribution density using the formula Mi = Ni/Si (I = 1, 2, 3....), where Mi is the clustering distribution density, Ni is the total number of POI data points that fall into the triangular irregular network, and Si is the area of the polygonal element.

## 4. Analysis of Overall Spatial Pattern for “Production–Living–Ecological” Space

### 4.1. Overall Spatial Pattern Analysis

**Figure 4:**
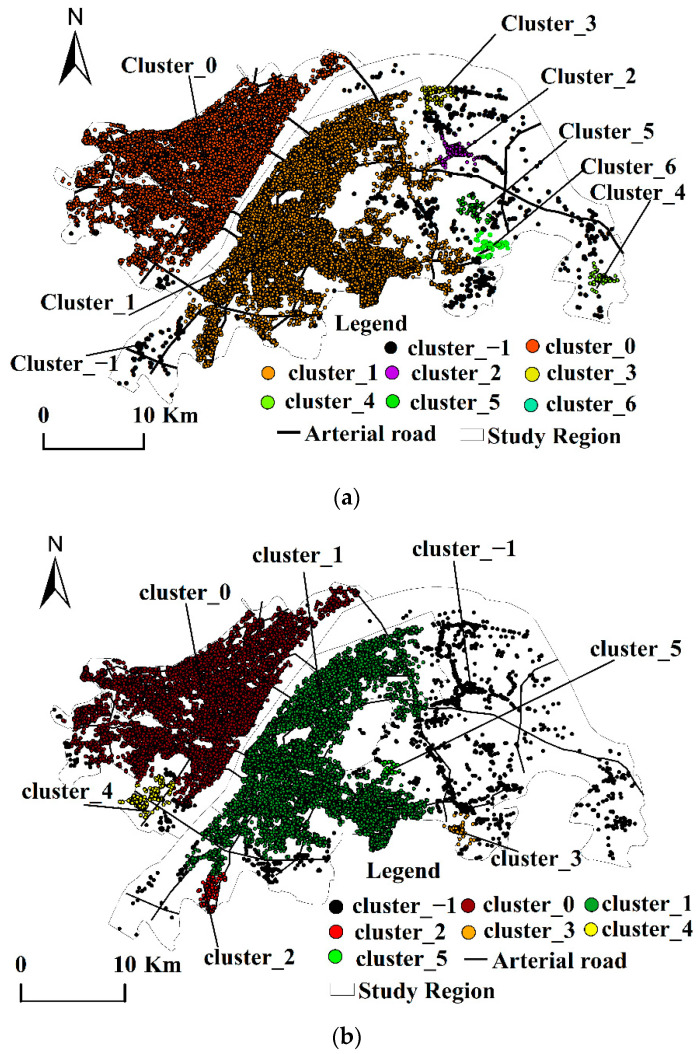

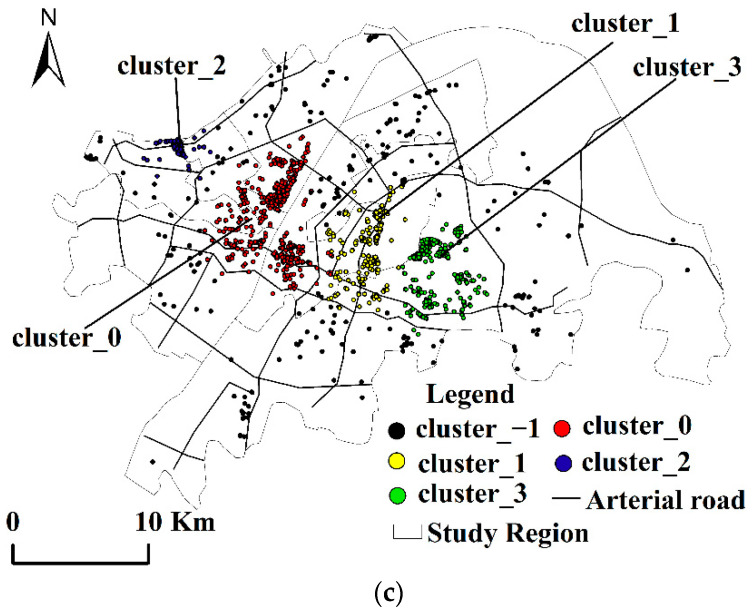
“Production–living–ecological” clustering spatial distribution pattern. (**a**) “Living” clustering spatial distribution pattern. (**b**) “Production” clustering spatial distribution pattern. (**c**) “Ecological” clustering spatial distribution pattern.

The values of ε and MinPts are determined after several experimental iterations. Seven clusters are formed by selecting the POI data for the production space, eight clusters are formed by the POI data for the living space, and five clusters are formed by the POI data for ecological space, as shown in Table 2. The overall space of the “production–living–ecological” space presents the following characteristics:

(1) Clustering presents spatial hierarchical distribution characteristics. In the living cluster (see Table 3a and Figure 4a), clusters 0 and 1 are larger in scale and exhibit a dual-core distribution pattern. The number of POI in cluster 0 reaches 134,697, which accounts for 49.45% of the total living POI, with a spatial distribution area of 198.11 km^2^. This cluster is concentrated in the Qiaokou, Hanyang, Jianghan, and Jiang’an Districts on the north bank of the Yangtze River. These districts are all located in the old city of Wuhan, which has many CBD (Central Business District) core areas, commercial and residential logistics areas, and government agencies, of which the Wuhan Municipal Government is located in Jiang’an District. The number of POIs in cluster 1 reaches 134,405, which accounts for 49.34% of all points, with a spatial distribution area of 241.90 km^2^, which is concentrated in the Wuchang and Hongshan Districts on the south bank of the Yangtze River. These districts are in the old city of Wuhan with many commercial and residential areas, lake parks, and school parks. Clusters 0 and 1 have a large service radius, which is the highest-level living function center in the city center of Wuhan and which shows significant spatial aggregation. Functional clustering not only provides rich life service functions for the internal space of the cluster location but also radiates this to other districts outside the administrative area. The other five clusters with relatively small scale and scope surround the larger clusters 0 and 1, which are distributed primarily in the east of the Hongshan District, the northern Qingshan District, and the southern Hongshan District. These have gradually become the secondary center of living functions. Clusters 0 and 1 have observed spatial spillover effects.

Among the production clusters (see Table 3b and Figure 4b), clusters 0 and 1 are the largest and are distributed on the north and south banks of the Yangtze River, which is similar to living clusters and shows a dual-core distribution pattern. The number of POI points in cluster 0 is 23,518, which accounts for 49.39% of the total production POI, with a spatial distribution area of 181.18 km^2^. These are concentrated in the four central urban areas on the north bank of the Yangtze River (the Qiaokou, Hanyang, Jianghan, and Jiangan Districts) and have become the highest-level production function centers in Wuhan. The number of POI points in cluster 1 is 21,555, which accounts for 45.26% of the point, with a spatial distribution area of 192.32 km^2^. These are concentrated in the Wuchang District and the southern part of the Hongshan District on the south bank of the Yangtze River. Clusters 0 and 1 show significant spatial aggregation phenomena and have obvious radiation effects on the other five smaller clusters (distributed in the east of Hongshan District), which gradually become the secondary centers of production function clustering.

Among the ecological clusters (see Table 3c and Figure 4c), from the perspective of quantity distribution, ecological clusters are relatively evenly distributed, which is distinct from living and production clusters. The largest cluster, 0, spans the north and south banks of the Yangtze River and is distributed in the Wuchang and Jiang’an Districts. The number of POI points is 648, with a spatial distribution area of 22.90 km^2^. Clusters 1 and 3 are formed around the East Lake Ecological Scenic Area. Cluster 1 has 252 POI points, with a spatial distribution area of 7.74 km^2^, and cluster 3 has 386 POI points, with a spatial distribution area of 16.16 km^2^.

(2) There is a strong polarization of the spatial distribution for living and production, and the clustering characteristics are similar. Production and living spaces are highly clustered and distributed in old urban areas on the north and south banks of the Yangtze River, which present integrated development trends with a high degree of overlap. The main reasons for this phenomenon are as follows. First, Wuhan is known as the “thoroughfare of nine provinces,” has a long history as the only sub-provincial megacity in the six central provinces, and is a core city in the Yangtze River Economic Belt. After several generations of effort, various infrastructure types and population and economic densities have improved over subsequent construction areas. Second, the interdependency between industries constitutes a virtuous market circle, which promotes the spatial agglomeration of various industries, deepens the degree of agglomeration, and accelerates the polarization of living and production spatial distributions, making them significantly overlap. As cities have become over-saturated in spatial developments, industries have gradually migrated to nearby unsaturated areas, which has created a spatial spillover effect. Third, policy orientations have brought elements such as administrative resources, population, and capital while promoting industrial and population agglomerations. The Hubei Provincial Government is in the Hongshan District, the Wuhan Municipal Government is in the Jiang’an District, and most of the university campuses are located in the Hongshan District.

(3) The accessibility of transportation significantly influences the layouts of the production and living spaces; however, the influence on the layout of the ecological space is not strong. The analysis of Figure 4a,b indicates that the living and production clusters are distributed in areas with good traffic accessibility, which are the largest and are located in core areas with developed traffic. The metro inner ring line, second ring line, third ring line, and main traffic arteries (such as Wuhan Avenue, Qintai Avenue, Youyi Avenue, Jiangcheng Avenue, Jiefang Avenue, Wuluo Road) pass through the functional areas where the living and production clusters are located. The Parrot Island Yangtze River Bridge, Wuhan Yangtze River Bridge, Wuhan Yangtze River Second Bridge, Wuhan Yangtze River Tunnel, and Erqi Yangtze River Bridge connect the living and production clustering areas on the north and south banks of the Yangtze River. Thus, the water and land transportation network is considered to be very developed. In addition, the living clusters (clusters 3, 5, 8, and 9) are distributed around the intersection of the Third Ring Road and Wu’e Expressway. Other smaller clusters are also distributed around the Third Ring Road, Wu’e Expressway, Qintai Avenue Intersection, and Wuhan Ring Expressway.

(4) The distribution of noise points is relatively concentrated, which shows the spatial aggregation potential of “production–living–ecological” functions. A total of 1050 noise points were generated in the living cluster, which accounts for 0.39% of the total. A total of 1716 noise points were generated in the production cluster, which accounts for 3.60% of the total. The living and production noise points are distributed primarily in the east and southwest corners of the Hongshan District. In general, these noise points are relatively scattered and isolated but clustered relatively locally. For example, there are relatively concentrated regions near the China University of Geosciences, Shimenfeng, Jiufeng, Yanxi Lake, Yandong Lake, and Wild Lake, which means these areas tend to become potential living or production clusters. There are 245 noise points generated in the ecological cluster, which accounts for 14.29% of the total. The noise points account for a larger proportion with a scattered and isolated distribution, which does not readily provide for future development. The overall analysis of the “production–living–ecological” cluster noise points gives a relatively scattered distribution that is isolated and attached to the vicinities of larger clusters. This shows the phenomenon of “large dispersion, small agglomeration.”

### 4.2. Analysis of Spatial Patterns for Element Layer

The DBSCAN algorithm is used to perform cluster analyses for each element in the element layer. Large differences in the number of POIs for each element cause the various elements to use distinct parameters. After multiple iterations, the specific MinPts values and their corresponding ε are selected, and the clustering density of each element is calculated based on the results. For the same target layer, clusters with a density greater than or equal to the mean of all clusters are regarded as areas with denser spatial distributions. These clustered areas are not only the hot spots where the target layer space gathers but are also important spatial nodes of the target layer spatial layout. Therefore, combined with the clustering results and densities, this paper identifies the important nodes for the spatial distributions of the “production–living–ecological” space. Based on the identification of the distributions of important spatial nodes, the “important spatial nodes-functional positioning” is analyzed according to the “Wuhan City territory spatial planning (2021–2035).”

#### 4.2.1. Analysis of Clustering Spatial Distribution Characteristics of Living Elements

**Figure 5:**
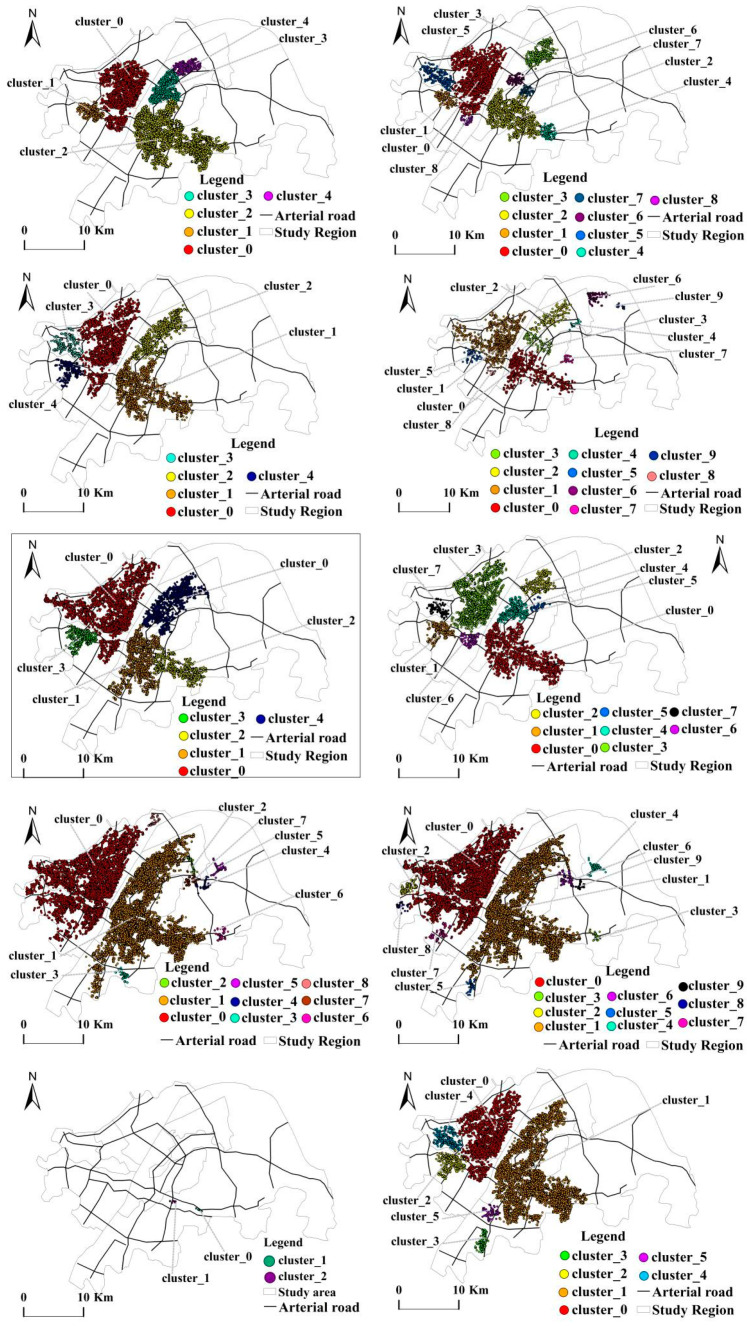
Spatial clustering distribution of the living elements.

The analysis in Table 4a–c and Figure 5 shows that most clusters are considered “important spatial nodes-function positioning” coincidences, but there are also dislocations or imbalances. The national economic center and regional financial center regions have become areas with the most important spatial nodes for various elements, which are followed by the commercial and logistics center regions. These three areas integrate urban living functions, such as residential, commercial, medical, and transportation facilities and public support services. These meet the requirements of living convenience, environment quality, and satisfaction indicators and provides efficient and livable urban living space. This is consistent with the establishment of the national economic center region as a key functional area leading to the modern service industry and is in line with regional finance. The construction of the central area into a financial clustering functional region coincides with the construction of the central area of commerce and logistics as a key functional area that carries commerce, consumption, and transportation.

There are, however, certain dislocations or imbalances. The two important spatial nodes for medical care elements are both distributed in the Jianghan and Hanyang Districts on the north bank of the Yangtze River, and there is the phenomenon of uneven development. The two important spatial nodes for sports and leisure elements are distributed in the Wangjiawan living area and near the Hongmiao overpass, which is far from many core areas of life and gives a dislocation phenomenon. The two important spatial nodes (clusters 6 and 9) for the public facilities elements are distributed near Jiaosha Road and Bajifu Street in the northern part of Hongshan District. This is an industrial area and does not match the functional positioning of living. Given these inconsistencies in the “important spatial nodes-functional positioning,” it is necessary to increase the layout of medical care on the south bank of the Yangtze River so that resources are developed in a balanced manner, increase the reasonable layout of sports and leisure facilities in living areas, strengthen the adjustment of public facilities in living function areas, and remove the industrial areas from living spaces in a reasonable and orderly manner.

#### 4.2.2. Analysis of Clustering Spatial Distribution Characteristics of Production Elements

**Figure 6:**
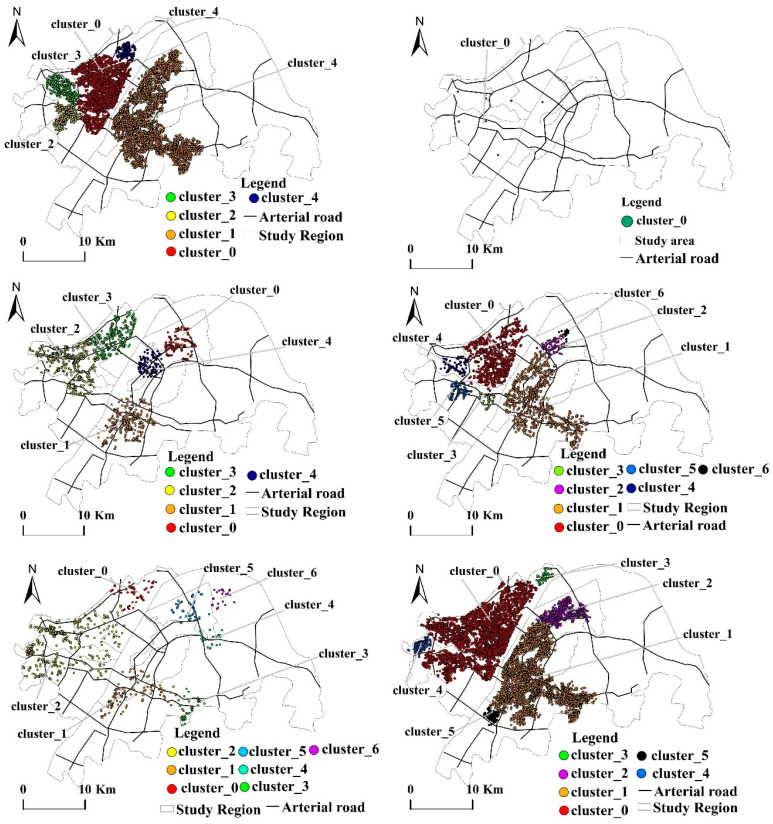
Spatial clustering distribution of production elements.

The analysis in Table 5a–c and Figure 6 shows that the important spatial nodes of a company’s corporate elements are concentrated in the core areas on the north and south banks of the Yangtze River. The important spatial nodes of the financial service elements are unevenly distributed and are primarily in the Jianghan District on the north bank of the Yangtze River. The important spatial nodes for the elements of the factory industrial park are scattered but distributed around the core areas of life in the Jianghan and Wuchang Districts and closely adjacent to the transportation hub. There is only one important spatial node of the car service element, which is clustered in the vicinity of transportation hubs, such as the Hanxi and Hankou Stations in the Qiaokou and Jianghan Districts. The important spatial nodes of the transportation elements are gathered in the Wuchang, Hongshan, Jianghan, and Hanyang Districts on the north and south banks of the Yangtze River. There is a coincidence phenomenon of “important spatial node-function positioning.” The national economic center region has become the area with the most production elements. The area is planned to deploy the city’s commercial service facilities in accordance with the four-level system of the “central business district, municipal business center, municipal business sub-center, and cluster business center”; cultivate a large-scale specialized commodity trading market; and strengthen Wuhan’s position as a regional commercial center. There are also certain dislocations or imbalances. The distribution of important spatial nodes of the financial service elements is unbalanced, does not match the planning, and needs adjustment. The important spatial nodes of the automobile service elements are singular, which is inconsistent with serving the social economy and giving play to the investment orientation of public service facilities.

#### 4.2.3. Analysis of Clustering Spatial Distribution Characteristics of Ecological Elements

**Figure 7:**
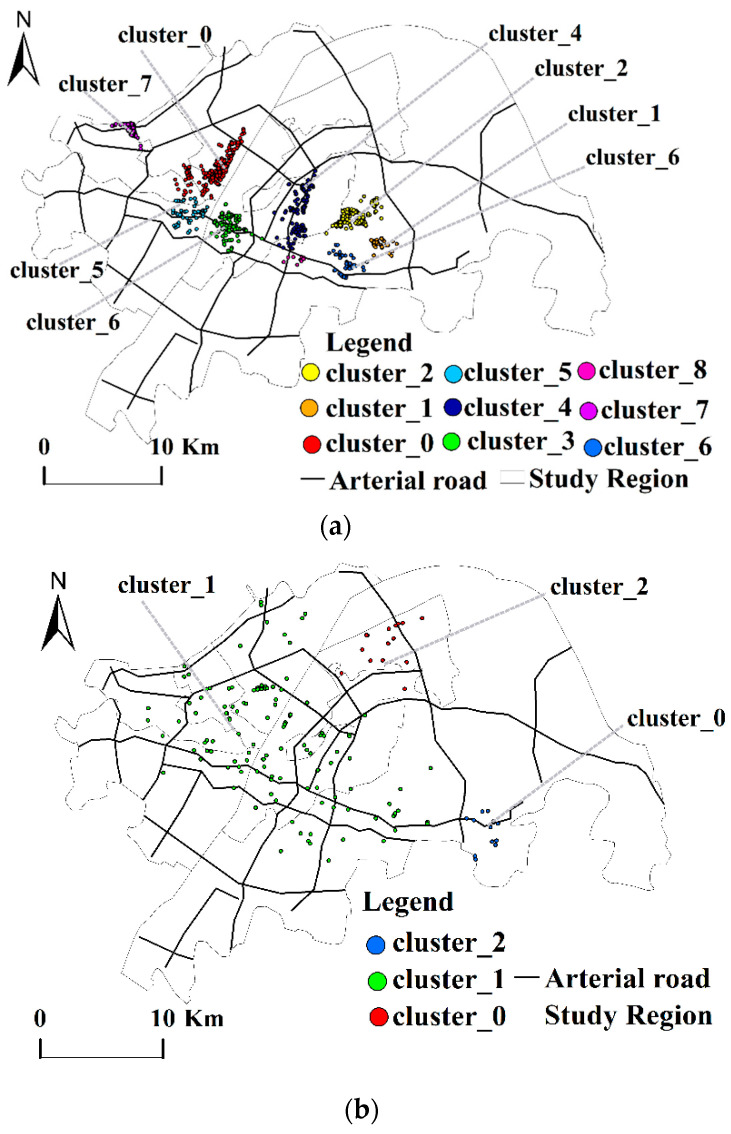
Spatial clustering distribution of the ecological elements. (**a**) Spatial clustering distribution of the parks and wetlands. (**b**) Spatial clustering distribution of the scenic spots.

The analysis in Table 6a–c and Figure 7a,b shows that important spatial nodes of the park green space elements are distributed near Jiufeng National Forest Park. The important spatial nodes of the scenic elements are near the Garden Expo Park on the north bank of the Yangtze River and the East Lake Scenic Park on the south bank. The important spatial nodes of ecology are in line with the plan of creating the “East Lake Green Heart World-class Urban Lake Model,” “Jiuzhen Mountain-Jiufeng Mountain East,” and “West Mountain Ecological and Cultural Corridor.”

## 5. Discussion

Based on POI big data and the DBSCAN cluster analysis method, this paper uses ArcGIS software to calculate cluster density, effectively identifies and analyzes the spatial pattern of “production–living–ecological” space in the central urban area of Wuhan (central Wuhan for short), discusses the causes of its formation, provides reference for the optimization of “production–living–ecological” space, and enriches its research field. The results suggest that POI big data and the DBSCAN cluster analysis method can effectively show the spatial pattern of “production–living–ecological” space in central Wuhan.

Traditional methods to obtain the spatial pattern of “production–living–ecological” space rely on the grouping of land use type and quantitative measurements of the index system, both of which are commonly used in the spatial pattern study of “production–living–ecological” space on medium, macroscopic, and patch scales. Depending strongly on the scale makes it difficult to reflect the dynamic nature of the space. Additionally, land use data being in the charge of relevant government departments results in difficult accessibility and poor timeliness; therefore, it is difficult to accurately depict the spatial pattern of “production–living–ecological” space by using these two methods. Compared with them, POI data are convenient and timely to obtain, and can dynamically and accurately reflect the distribution pattern of “production–living–ecological” space in the central urban area of Wuhan. Just because of these advantages, POI is widely used in the analysis of spatial patterns of urban facilities, evaluation of urban living environments, and the division of urban functional space. The spatial pattern analysis of urban facilities uses spatial statistical analysis methods, such as density estimation, to characterize the aggregation and dispersion of a certain urban element. The evaluation of urban living environments uses cross-type POI data to comprehensively evaluate the convenience brought by urban facilities to residents in living services and objectively describe the interaction between human and land elements. The division of urban functional space by measuring the spatial density of urban entity POI data and dividing urban space into one or more high POI density value areas objectively reflects the spatial pattern of man–land system elements. In addition, to explore the law of human, traffic, information, and financial flow hidden in POI data is also an important trend of research. Therefore, making use of the point element data form of POI can not only reveal the spatial pattern characteristics of man-land system elements but also show the internal mechanism of sustainable development of the man–land system.

“Production–living–ecological” space is a more comprehensive method of territorial space division, and it is also a physical space with rich semantics. Its goal is to create balanced and sustainable development for different types of space and to improve the livability of the city and the quality of life of residents, which is consistent with Pablo F et al. [43]. “Production–living–ecological” space has the attributes of area, volume, distance, etc., but also endows the physical and socio-economic dimensions of geographical entities, reflecting the physical space carrying the three functions of production, life, and ecology, so it is also regarded as a semantically rich physical space [44,45]. In the future, rich geographic information will be combined with humanistic perspectives, such as production, live, and ecology, to support urban planning and improve the quality of urban space.

This paper compares the research results with the overall planning of Wuhan’s territorial space, analyzes the positioning of important spatial nodes and functions, and finds that there is a dislocation or imbalance of “important spatial nodes-functional positioning”. In the living space, it is necessary to increase medical care units to ensure the balanced development of the north and south banks of the Yangtze River, adjust the layout of public facilities reasonably, and guide living functions in an orderly manner to develop towards the periphery of a high-concentration area in order to avoid social problems, such as traffic jams and high population density caused by an excessive concentration of people. For the production space, the following ideas are suggested: develop a large-scale specialized commodity trading market continuously to strengthen Wuhan’s status as a regional commercial center; distribute the financial services elements evenly; strengthen the role of transportation service spaces in social and economic development; strengthen the production services function of “multi-cluster” centers; and avoid increasing the pressure in urban core areas in terms of population, transportation, resources, and the environment. In the ecological space, continuously building the ecological corridors and important ecological scenic spots is suggested.

## 6. Conclusions

This paper constructs a POI classification system based on the understanding of the formation mechanism and definition of the urban “production–living–ecological” space. The DBSCAN clustering algorithm is used to calculate the clustering density with the ArcGIS software to study the spatial patterns of “production–living–ecological” space in the central urban area of Wuhan. The research conclusions are as follows:

1. Overall spatial pattern: The living and production spatial distributions have strong spatial hierarchical characteristics with significant polarization, while the ecological spatial distribution is more balanced. (1) The living and production spaces form two large clusters that are in the core areas of the north and south banks of the Yangtze River with a high degree of overlap. Several small clusters are distributed around the two large clusters, with a strong spatial spillover effect. (2) There are relatively few living and production noise points, but the local distribution is concentrated and can easily become a potential development area. (3) The accessibility of transportation plays an important role in promoting the distribution of the “production–living–ecological” space. (4) In the future, it will be necessary to rationally guide the expansion of living and production functions, strengthen the diversified gathering center, and alleviate the pressure on the population and resources in core areas. The ecological functions should go deep into the main urban areas in the future, establish ecological corridors and urban air ducts that connect the inside and outside of the city, highlight urban scenic areas, improve the urban heat island effect, enrich the green space landscape, form a well-structured park green space system, and reduce the noise points of ecological functions.
2. Spatial pattern of elements: (1) The important spatial nodes of most of the living and production elements are distributed in the core areas of life and production functions, and the important spatial nodes of the ecological elements are distributed in parks, green areas, and urban core scenic areas. (2) Most of the important spatial nodes for living and production are consistent with the overall planning of Wuhan, but there are certain differences in the distributions of important spatial nodes for some elements. The important spatial nodes of the ecological elements are consistent with the ecological planning of Wuhan. (3) In the living space, retail monopolies, life services, and public squares have greater impacts on the formation and impact of the space. In the production space, the elements of corporate enterprises, financial services, and factories have greater impacts. In the ecological space, scenic spots have a greater impact on regulation.

The conclusion shows that, based on POI big data, with the help of ArcGIS software and the DBSCAN cluster analysis method, the disadvantages of traditional data are overcome, and the spatial distribution pattern of “production–living–ecological” space in the central urban area of Wuhan is effectively characterized. However, POI point elements lack geographic entity area attributes, socioeconomic and human factors, and other attribute information, which hinders POI data from in-depth exploration of the law of human–earth system coupling, which will be the direction of continued research after this paper. In addition, this paper will also improve the DBSCAN method to improve the local search ability, enhance the diversity of clusters, and optimize the process of cluster merging.

## Figures and Tables

**Table 1:** Urban “production–living–ecological” space POI data classification system.

Target Layer	Criterion Layer	Element Layer	Industrial Classification	Number of POIs
Production space	Business space	Corporate enterprises	Advertising, decoration, construction companies, etc.	22,957
		Financial services	Banks, insurance, securities companies, etc.	5561
	Industrial space	Factory	Factories, workshops, etc.	1162
		warehousing logistics	Warehouses, logistics, rail stations, etc.	10
		Auto services	Automobile sales, maintenance companies, etc.	4926
	Transportation space	Transportation	Subway stations, bus stations, parking lots, airports, railway stations, wharfs, etc.	12,920
Living space	Habitable space	Residential buildings	Villas, urban residential areas, and rural homesteads	13,216
	Service space	Retail monopolies	Retail stores, specialty stores, convenience stores, gift shops, etc.	68,731
		Supermarket shopping	Comprehensive shopping markets, malls, etc.	31,453
		Hotel catering	Casual restaurants, hotels, etc.	60,130
	Public space	Life services	Beauty salons, photography shops, funeral facilities, etc.	52,382
		Medical treatment	Hospitals, veterinary practices, etc.	10,994
		Science and education	Schools, museums, research institutions, etc.	15,546
		Sports and leisure	Sports and entertainment venues, etc.	8097
		Communal facilities	Public toilets, news kiosks, etc.	2792
		Public squares	Public squares	129
	Management space	Government agencies	Government agencies, etc.	8950
Ecological space	Green space	Parks and wetlands	Parks, zoos, botanical gardens, wetlands, etc.	168
		Scenic spots	Scenic spots, temples, etc.	1592

**Table 2:** The “production–living–ecological” spatial clustering parameters.

	MinPts (Number)	ε (km)	Evaluation Coefficient	Clusters (Number)
Production space	95	1	0.111	7
Living space	105	1	0.345	8
Ecological space	30	1.5	0.416	5

**Table 3:** Clustering density of the “production-living-ecological” space (Number/km^2^).

**(a) Clustering Density of the Living Space (Number/km^2^)**								
**Cluster Type**	**Cluster _0**	**Cluster _1**	**Cluster _2**	**Cluster _3**	**Cluster _4**	**Cluster _5**	**Cluster _6**	**−1 (Noise Points)**
Number of POIs	134,697	134,405	1129	290	367	206	254	1050
Cluster area	198.11	241.90	2.06	3.34	1.42	1.96	1.08	-
**(b) Clustering Density of the Production Space (Number/km^2^)**								
**Cluster Type**	**Cluster _0**	**Cluster _1**	**Cluster _2**	**Cluster _3**	**Cluster _4**	**Cluster _5**	**−1 (Noise Points)**	
Number of POIs	23,518	21,555	229	180	366	57	1716	
Cluster area	181.18	192.32	2.88	0.75	3.874	1.16	-	
**(c) Clustering Density of the Ecolog** **ical ** **Space (Number/km^2^)**								
**Cluster Type**			**Cluster _0**	**Cluster _1**	**Cluster _2**	**Cluster _3**	**−1 (Noise Points)**	
Number of POIs			648	252	184	386	245	
Cluster area			22.90	7.74	1.45	16.16	-	

**Table 4:** Spatial distribution characteristics of living elements.

**(a) Living Element Layer Clustering Parameters**													
**Element**			**101**	**102**	**103**	**104**	**105**	**106**	**107**	**108**	**109**	**110**	**111**
MinPts			110	100	200	120	80	80	90	80	30	6	90
ε			1	1	1	1	1	1	1	1	1	1	1
**(b) Cluster Density of Living Elements**													
**Element**	**Cluster _0**	**Cluster _1**	**Cluster _2**	**Cluster _3**	**Cluster _4**		**Cluster _5**	**Cluster _6**	**Cluster _7**	**Cluster _8**		**Cluster _9**	**Mean**
101	**62.67**	40.23	**73.54**	40.76	39.26		-	-	-	-		-	51.29
102	**262.45**	**185.62**	112.57	102.45	**179.29**		90.72	81.62	**133.30**	59.71		95.12	130.28
103	**146.94**	**127.43**	84.75	**124.24**	106.31		-	-	-	-		-	117.93
104	221.60	170.33	**263.05**	163.72	**469.77**		161.93	225.58	-	-		-	239.43
105	**158.78**	**146.79**	**104.65**	93.75	54.79		**128.05**	42.58	74.81	55.40		-	95.51
106	**58.19**	47.81	49.41	42.01	**58.88**		-	-	-	-		-	51.26
107	**49.22**	**64.82**	40.01	**48.97**	37.74		38.54	-	-	-		-	46.55
108	35.14	**53.38**	36.30	40.55	36.09		**70.78**	41.60	33.04	-		-	43.36
109	16.06	15.43	12.69	15.42	**43.26**		20.87	**27.07**	17.95	**35.25**		**37.15**	24.12
110	792.81	28.25	-	-	-		-	-	-	-		-	410.53
111	**48.76**	37.82	**43.04**	35.95	29.78		**38.46**	33.59	38.06	40.70		-	38.46
**(c ) Important Spatial Nodes in the Distribution of the Living Space.**													
**Element**				**Larger Cluster**			**Distribution Area**						
101				Clusters _0 and 2			National Economic Center, Regional Financial Center, and National Science and Technology Innovation Center						
102				Clusters _0, 1, 4, and 7			National Economic Center, Regional Financial Center, and National Science and Technology Innovation Center						
103				Clusters _0, 1, and 3			National Economic Center, Regional Financial Center, and Business Logistics Center						
104				Clusters _2 and 4			National Economic Center and Regional Financial Center						
105				Clusters _0, 1, 2, and 5			National Economic Center, Regional Financial Center, National Science and Technology Innovation Center, Trade and Logistics Center, and International Exchange Center						
106				Clusters _0 and 4			National Economic Center, Regional Financial Center, and Commercial and Logistics Center						
107				Clusters _0, 1, and 3			National Economic Center, Regional Financial Center, and National Science and Technology Innovation Center						
108				Clusters _1 and 5			Commerce and Logistics Center						
109				Clusters _4, 6, 8, and 9			-						
110				Cluster _0			-						
111				Clusters _0, 2, and 5			National Economic Center, Regional Financial Center, and Commercial and Logistics Center						

Description: Residential buildings (101), Retail monopolies (102), Supermarket shopping (103), Hotel Catering (104), Life services (105), Medical treatment (106), Science and education (107), Sports and leisure (108), Communal facilities (109), Public squares (110), and Government agencies (111). Bold numbers indicate that the cluster density is greater than or equal to the mean of all clusters.

**Table 5:** Spatial distribution characteristics of production elements.

**(a) Production Element Layer Clustering Parameters**								
**Element**	**201**	**202**		**203**	**204**	**205**		**206**
MinPts	120	50		20	6	160		100
ε	1	1		2	1	2		1
**(b) Cluster Density of the Production Elements**								
**Element**	**Cluster _0**	**Cluster _1**	**Cluster _2**	**Cluster _3**	**Cluster _4**	**Cluster _5**	**Cluster _6**	**Mean**
201	**72.86**	**73.19**	45.17	48.40	**69.18**	55.40	-	60.70
202	**37.89**	27.70	28.04	**41.18**	27.66	28.70	**58.02**	35.60
203	**14.75**	**13.55**	**15.89**	8.31	3.50	4.53	4.13	9.24
204	0.09	-	-	-	-	-	-	0.09
205	27.63	26.65	**63.54**	20.44	25.99	-	-	32.85
206	**62.08**	**47.74**	45.41	41.14	34.03	-	-	46.08
**(c) Important Spatial Nodes in the Distribution of the Production Space**								
**Element**	**Larger Cluster**		**Distribution Area**					
201	Clusters _0, 1, and 4		National Economic Center, Regional Financial Center, National Science and Technology Innovation Center, Trade and Logistics Center, and International Exchange Center					
202	Clusters _0, 3, and 6		National Economic Center, Regional Financial Center, and Commercial and Logistics Center					
203	Clusters _0, 1, and 2		National Economic Center and Commercial and Logistics Center					
204	Cluster _0		National Economic Center					
205	Cluster _2		Commerce and Logistics Center					
206	Clusters _0 and 1		National Economic Center, Regional Financial Center, National Science and Technology Innovation Center, and Commercial and Logistics Center					

Description: Corporate enterprises (201), Financial services (202), Factory (203), Warehousing logistics (204), Auto services (205), Transportation (206). Bold numbers indicate that the cluster density is greater than or equal to the mean of all clusters.

**Table 6:** Spatial distribution characteristics of ecological elements.

**(a) Ecological Element Layer Clustering Parameters**										
**Element**	**301**					**302**				
MinPts	6					20				
ε	3					1				
**(b) Cluster Density of Ecological Elements**										
**Element**	**Cluster _0**	**Cluster _1**	**Cluster _2**	**Cluster _3**	**Cluster _4**	**Cluster _5**	**Cluster _6**	**Cluster _7**	**Cluster _8**	**Mean**
301	2.28	2.30	4.04							2.87
302	47.19	20.35	76.39	39.34	39.18	23.12	23.11	225.63	36.11	58.94
**(c) Important Spatial Nodes in the Distribution of the Ecological Space**										
**Element**	**Larger Cluster**			**Distribution Area**						
301	Cluster _2			Jiufeng National Forest Park						
302	Clusters _2 and 7			Garden Expo Park and East Lake Scenic Park						

Description: Parks and wetlands (301), Scenic Spots (302). Bold numbers indicate that the cluster density is greater than or equal to the mean of all clusters.

## Data Availability

No new data were created or analyzed in this study. Data sharing is not applicable to this article.
